# Supplementary figures and images for: LIN-35 is necessary in both the soma and germline for preserving fertility in Caenorhabditis elegans under moderate temperature stress
Source: PLoS One. 2023 Jun 9;18(6):e0286926. doi: 10.1371/journal.pone.0286926 (PMC10256190; doi:10.1371/journal.pone.0286926)

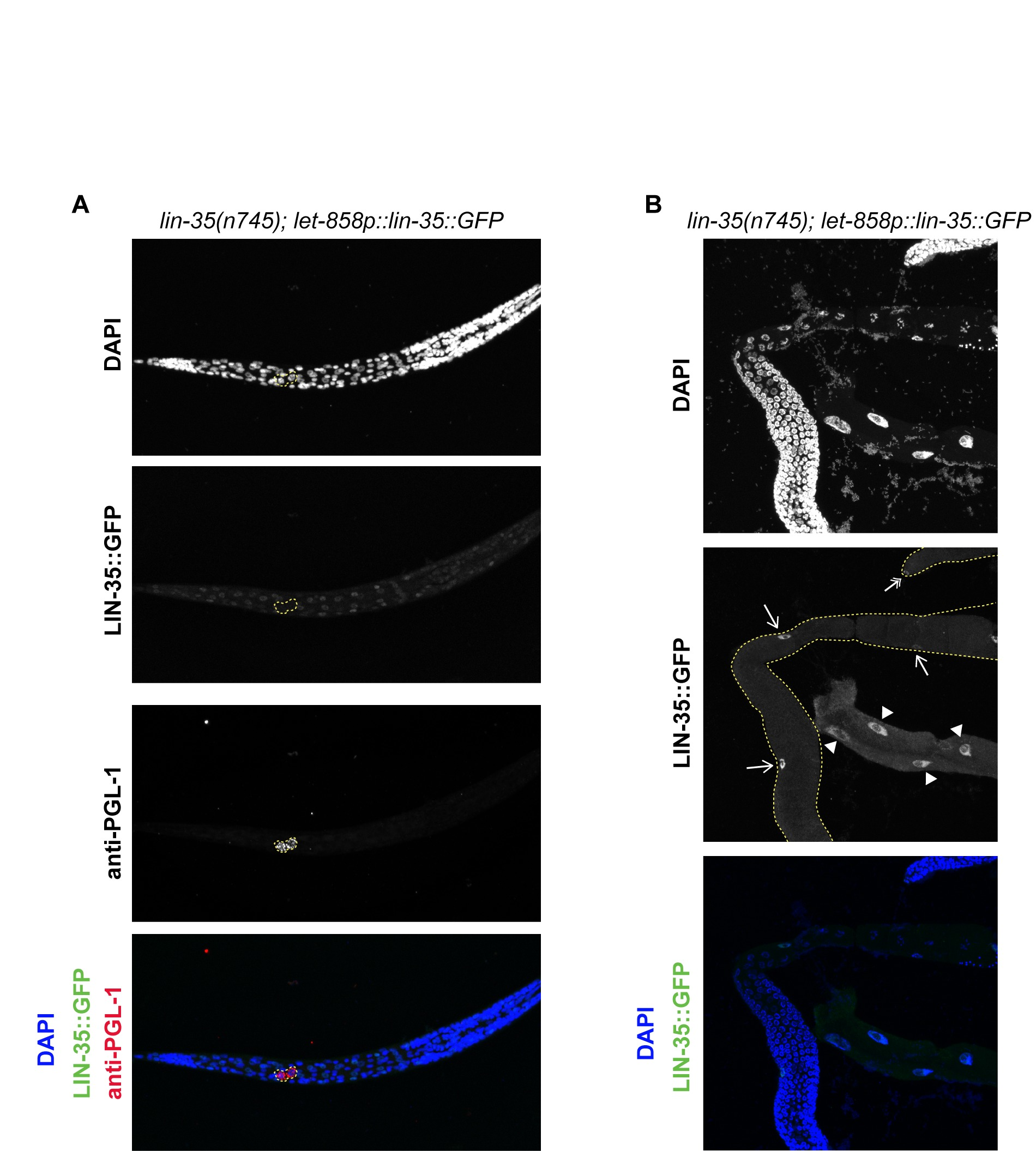

Supplement: S1 Fig — (A) L1 animals at 20°C display broad expression of LIN-35::GFP in somatic tissues, but not in the the two primordial germ cells Z2/Z3 (shown by staining with anti-PGL-1). Z2/Z3 outlined with a yellow-dashed line. (B) Images of adult gonad tissue beside adult intestine show expression of LIN-35::GFP in the somatic distal tip cell (double-arrow), somatic gonad sheath cells (arrows), and intestinal cells (arrow heads) but not in either mitotic or meiotic germ cells. The gonad tissues are surrounded by yellow-dashed lines. (TIF) [file pone.0286926.s001.tif]

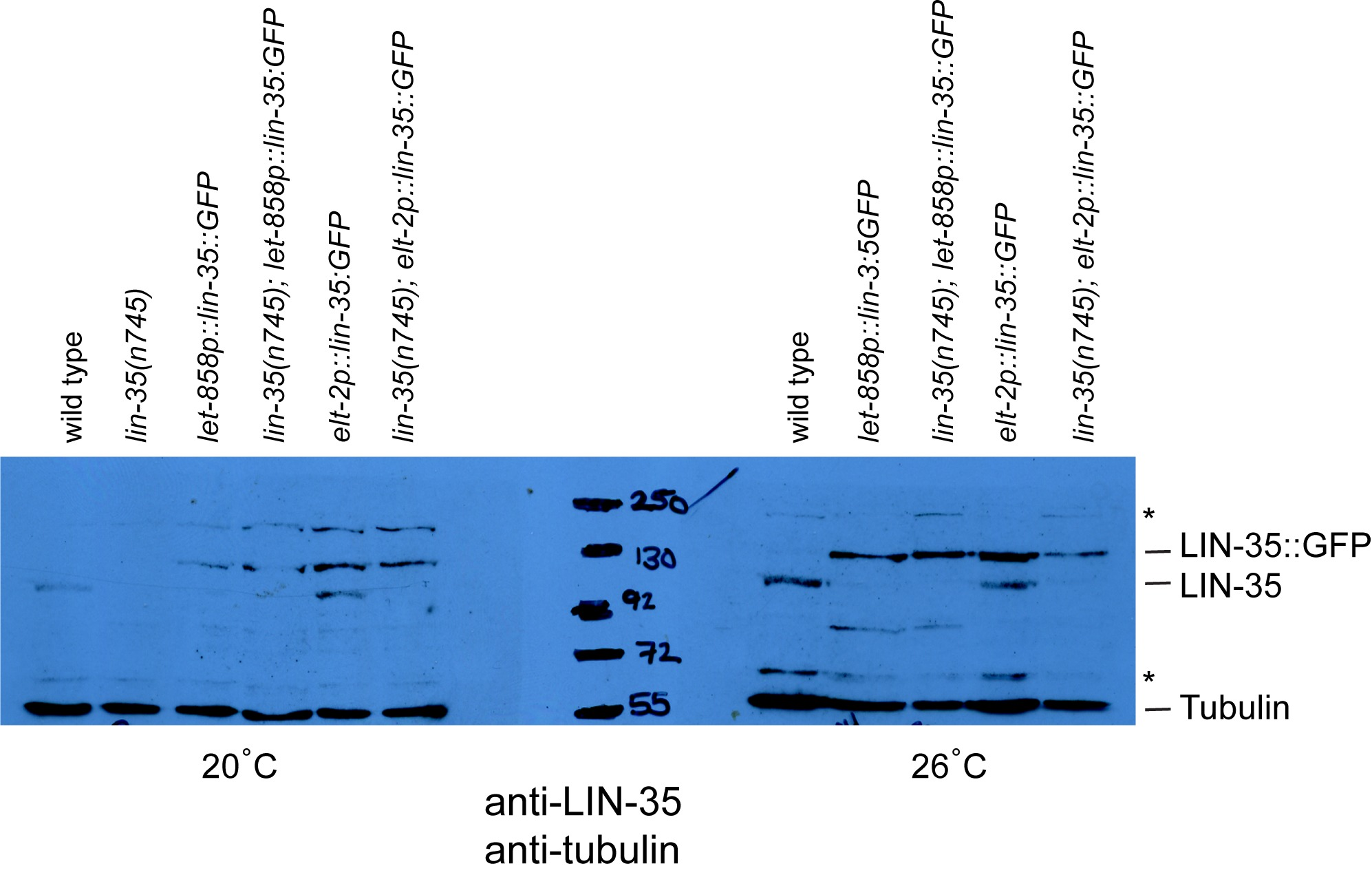

Supplement: S2 Fig — Western blot using anti-LIN-35 antibodies shows expression of both the wild-type LIN-35 protein and LIN-35::GFP tagged protein expressed from somatic transgenes in animals grown at either 20°C or 26°C. In wild-type worms expressing the let-858p::lin-35::GFP transgene, only LIN-35::GFP protein can be seen, while in wild-type worms containing the elt-2p::lin-35::GFP transgene, both LIN-35 and LIN-35::GFP protein can be seen. Anti-Tubulin was used on the same blot as a loading control. * non-specific background bands. (TIF) [file pone.0286926.s002.tif]
